# Study of the Antibacterial Activity of Rich Polyphenolic Extracts Obtained from *Cytisus scoparius* against Foodborne Pathogens

**DOI:** 10.3390/antibiotics12111645

**Published:** 2023-11-20

**Authors:** Lorena G. Calvo, Aly Castillo, Rosa-Antía Villarino, José Luis R. Rama, Ana G. Abril, Trinidad de Miguel

**Affiliations:** 1 Department of Microbiology and Parasitology, Universidade de Santiago de Compostela, E-15782 Santiago de Compostela, Spain; lorenagomez.calvo@usc.es (L.G.C.); rosaantia.villarino@rai.usc.es (R.-A.V.); joseluis.rodriguez.rama@usc.es (J.L.R.R.); anagonzalez.abril@usc.es (A.G.A.); 2 i-Grape Laboratory, Emprendia, Campus Vida, E-15782 Santiago de Compostela, Spain; alyjesus.castillo.zamora@usc.es; 3 Laboratory of Research and Development of Analytical Solutions (LIDSA), Department of Analytical Chemistry, Nutrition and Food Science, Universidade de Santiago de Compostela, E-15782 Santiago de Compostela, Spain

**Keywords:** polyphenols, antimicrobials, biofilms

## Abstract

Natural extracts containing high polyphenolic concentrations may act as good antimicrobials for their antibacterial and antibiofilm activity. The present research characterizes two hydro-organic extracts with high polyphenolic content, obtained from the shrub *Cytisus scoparius* as antipathogenic candidates. As a result of their own composition, both extracts, LE050 and PG050, have shown pronounced bioactivities with potential uses, especially in agricultural, livestock production, food manufacturing, and pharmaceutical industries. Polyphenolic compounds were extracted by using adjusted hydro-organic solvent mixtures. These extracts’ in vitro antimicrobial activity was evaluated on Gram-positive and Gram-negative pathogenic bacteria, giving special attention to those involved in food contamination. Due to this, the biofilm dispersion was assessed on *Listeria monocytogenes*, *Staphylococcus aureus* and *Pseudomonas aeruginosa*. The extracts showed antimicrobial activity against the pathogenic species tested, presenting IC50 values between 0.625–20% *v*/*v*. Different behaviors have been detected between both extracts, probably linked to their distinct polyphenol composition, being LE050 extract the one with most promising bioactive applications. Finally, the results from the biofilm dispersion assays reveal that the extracts exhibit a good antibiofilm activity against the pathogenic bacteria tested.

## 1. Introduction

Polyphenols are the most abundant secondary metabolites present in the plant kingdom. They represent a large and diverse group of molecules including two main families, the flavonoids and the non-flavonoids. Their diverse composition provides them with numerous therapeutic properties, including the regulation of pH and metabolism, antioxidative defense, as well as antimicrobial and anti-inflammatory activities [1].

In recent years, antimicrobial resistance (AMR) has become a serious health problem and a major global issue [2]. Infections caused by antimicrobial-resistant bacteria are difficult or even impossible to treat and they are becoming increasingly common. AMR not only depends on the misuse of antibiotics in human treatments. In fact, the use of antimicrobials in livestock feed and as a conservative method by the food industry has been a major factor in the emergence and spread of antimicrobial resistance [3].

Foodborne diseases are caused by the consumption of food contaminated with pathogenic microorganisms and their toxins. According to a report by the World Health Organization, it is estimated that there is a global outbreak of 600 million foodborne diseases yearly, which results in 420,000 deaths [3]. Among the different food contamination causes, the main reason for foodborne diseases are bacteria (66%) [4], such as *Campylobacter jejuni*, *Bacillus cereus*, *Clostridium perfringens*, *Listeria monocytogenes*, *Escherichia coli*, *Salmonella* spp., *Staphylococcus aureus*, or *Yersinia enterocolitica* [5].

Despite the considerable effort to improve production technologies, manufacturing, hygiene standards, and correct consumers education, spoilage and foodborne pathogenic microorganisms still spread and cause huge economic losses [6]. Due to this, the demand for fresh-minimally processed and ready-to-eat foods by the consumers makes new routes emerge for the pathogens to spread. At the same time, changes in the society that involve ecological concerns and a growing demand for more environmentally friendly food, have led researchers, consumers, and food manufacturers, to direct their attention to finding new natural sources of antimicrobials for food preservation [7].

The antimicrobial potential of polyphenols found in vegetable foods and medicinal plants has been extensively explored against a broad spectrum of microorganisms [8]. Among polyphenols, flavan-3-ols, flavonols, and wine tannins have been the most deeply studied and received great attention due to their wide spectrum and higher antimicrobial activity. Most of them are able to suppress microbial pathogenic factors through inhibition of biofilm formation, reduction of host ligands adhesion, and neutralization of bacterial toxins [9]. Polyphenols possess a wide range of mechanisms of action on bacteria, being membrane damage the most prominent. The OH group of the phenolic compounds causes membrane cell disruption through hydrogen bonding interactions [10,11]. The presence and position of OH functional groups are relevant to the antibacterial activity of the polyphenols [12].

Lipophilic character of polyphenols is directly related to their antimicrobial activity, possibly due to their potential interactions with the cell membrane [13]. The ability to penetrate the membrane and interact with the cell compounds induces irreversible damage, causing cell death and intracellular content liberation [14].

Linked to the above-mentioned mechanism of action, gram-negative bacteria are generally more resistant to antimicrobial agents than gram-positive ones [15]. This may be due to the effectiveness of the outer membrane in slowing down the passage of molecules into the cell [16], together with the occurrence of efflux pumps, which play an important role in the AMR [17].

Besides their ability to limit the development of pathogenic microorganisms, polyphenols participate in biofilm dispersion and reduction. A biofilm is a sessile form of bacterial existence on solid surfaces or liquid interfaces, wherein bacteria envelop themselves with a self-generated biofilm matrix comprising intercellular polysaccharides, proteins, and extracellularly released nucleic acids [18]. This protective structure facilitates microorganisms’ self-establishment and spreading, attaching them to a surface and endowing their metabolic maintenance and quorum sensing [19]. Additionally, biofilms act as defensive structures against antibiotics. Their protective effect involves reducing the penetration of antimicrobial agents into the deeper layers of biofilms, capturing positively charged molecules through the extracellular polymeric biofilm matrix, and the capacity of biofilm matrices to concentrate bacterial enzymes that can deactivate antibiotics, among other mechanisms [20].

Most of the principal foodborne pathogenic bacteria are well known for their biofilm formation. Some of them are major contaminants in food manufacturing and livestock production. *Listeria monocytogenes* is a human foodborne intracellular pathogen known for its resilience to various stress conditions. The correlation between its resistance to oxidative stress and biofilm formation makes *L. monocytogenes* exceptionally challenging to manage throughout the entire food chain, spanning from production to storage and consumption [21]. *Pseudomonas aeruginosa* is an opportunistic Gram-negative pathogen, able to attach to both abiotic and biotic surfaces. Several *P. aeruginosa*’s genes involved in biofilm formation have been studied for their role in quorum sensing, suggesting that biofilms are important for the development and pathogenesis of this bacterial species [22].

*Cytisus scoparius* is a perennial leguminous shrub ordinarily distributed in the northern areas of Europe. *Cytisus* spp. mainly grow in disturbed or neglected areas and its pruning is one of the measures followed to fight against rapidly spread forest fires [23]. In botanical-medical treatments, this plant is used for diuretics, hypnotics, and sedatives [24]. Furthermore, it can contribute to the treatment of diabetes and liver diseases. It has also been found to have hypotensive activity and estrogenic effect [25]. Most of the biological activities detected in this plant may relate to its antioxidant natura and its active constituents. Previous studies have been done on *C. scoparius* phenolic profile and antimicrobial activity, showing promising results [26].

The present study evaluates the in vitro antimicrobial activity of hydro-organic polyphenol-rich extracts of *C. scoparius*. Bacterial viability and biofilm dispersion have been assessed in the presence of the extracts, with special attention given to foodborne pathogens. The aim of this work is to help the fight against bacterial resistances, by promoting healthy and environmentally friendly livestock raising and food production.

## 2. Results

The present study reports the assessment of the antimicrobial and antioxidative capacity of two polyphenol rich extracts to be used as potential antimicrobial agents.

### 2.1. Extract Characterization, Total Polyphenol Index (TPC), and Antioxidative Activity Determination

The hydro organic solvent mixtures used to obtain extracts LE050 and PG050 were ethyl lactate: water 50:50 (*v*/*v*) and propylene glycol: water 50:50 (*v*/*v*) respectively. During the polyphenol extractive process, this proportion of solvent and water changed due to the water releasing from the plant cells, leading to a final aqueous volume of 73.6% ± 0.1 for LE050 and 71.9% ± 0.1 for PG050. In order to calculate the percentage of solids—remaining insoluble fraction—present in the extracts, the extracts moisture was removed and the values for the remaining solids measured were 4.1% (*w*/*v*) and 3.7% (*w*/*v*) for LE050 and PG050, respectively.

In order to evaluate the potential effect of solvent polarity as polyphenol extractants the total polyphenol index (TPC) of the extracts was obtained using the Folin–Ciocalteu method. The results reveal a concentration of 4449 mgGAE/L (GAE: gallic acid equivalent) for LE050 and 1003 mgGAE/L for PG050.

The result of the antioxidative activity using the 2,2-diphenyl-1-picrylhydrazyl (DPPH) reagent was 1.44 mmolTE/L for LE050 extract and 0.77 mmolTE/L for PG050 extract.

### 2.2. LC-MS/MS Characterization of the Extracts

**Figure 1:**
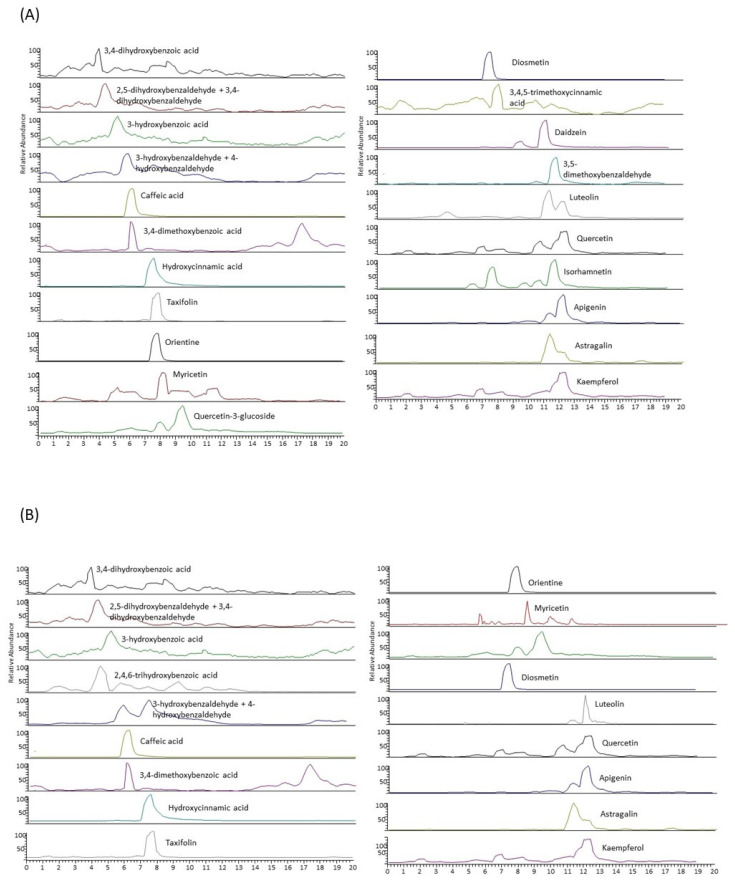
Reconstructed chromatogram for the target polyphenols detected in LE050 (**A**) and PG050 extracts (**B**).

**Figure 2:**
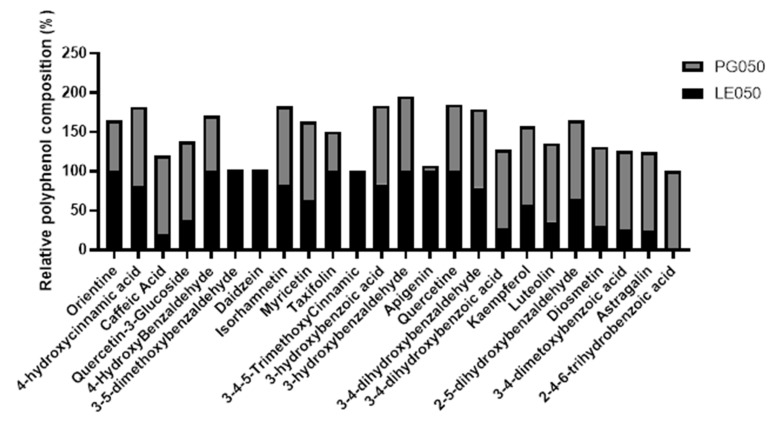
Polyphenolic characterization of *C. scoparius’* LE050 and PG050 extracts. Normalized concentration of target identified polyphenols are represented as relative percentages.

Characterization of the polyphenolic extracts was performed by liquid chromatography (LC-MS/MS). Chromatograms for the target analytes of the extracts and individual target polyphenol values are normalized and expressed as relative concentrations in Figure 1 and Figure 2, respectively.

Significative differences were observed on the composition of both extracts (*p* < 0.05). Twenty-four different polyphenols were detected; however, just only twenty of them are presented in both extracts. LE050 extract posses a higher diversity of target polyphenols, although 2-4-6-trihydrobenzoic acid was not identified in this extract. As Figure 2 shows, PG050 extract possess twenty-one of the total polyphenols, but no 3-5-dimethoxybenzaldehyde, daidzein, and 3-4-5-trimethoxycinnamic were detected. Moreover, very low concentrations of apigenin were observed. However, PG050 extract possess higher relative amounts of phenolic acids, such as caffeic acid, 3-4-dimetoxybenzoic acid, 3-4-dihydroxybenzaldehyde, and 2-4-6-trihydrobenzoic acid. Flavonoids are the most common polyphenols identified in the extracts, being flavonols, such as quercetin, kaempferol, myrciting, and isorhamnetin those with higher abundances. Only one isoflavonoid was identified—daidzein, which is only present in the LE050 extract. This extract also presents higher amounts of hydroxybenzaldehydes in comparison with PG050 extract. These values suggest that solvents play a main role in the polyphenol’s bioaccessibility and probably will shape the bioactivities of the extracts.

### 2.3. Antimicrobial Activity

The antimicrobial activity of the extracts against foodborne pathogens was carried out following the alamarBlue viability protocol. EUCAST inhibitory assay recommendations were followed.

**Figure 3:**
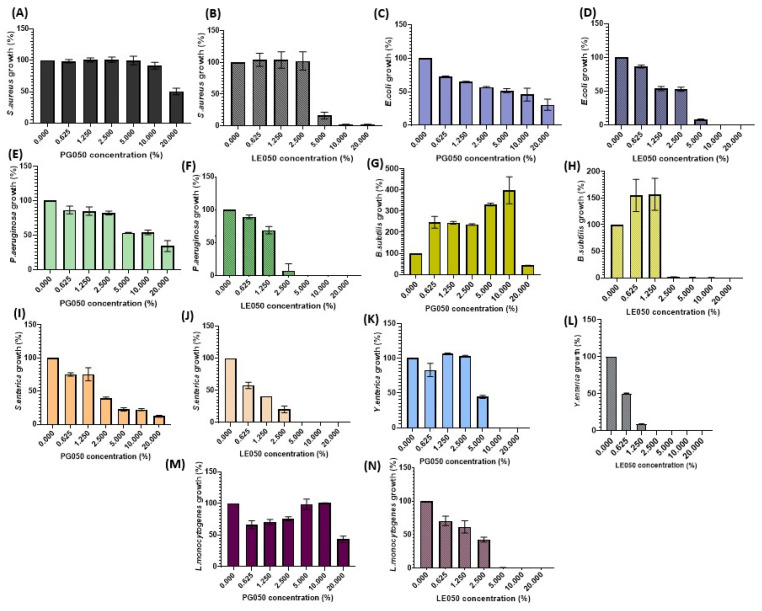
Bacterial growth tendency under different *C. scoparius* PG050 and LE050 extract’s concentration. Experiments were performed in triplicate and three times. Data are presented as percentages of growth with respect to the bacterial concentration achieved in the absence of extract and standard deviation of the replicates is indicated. (**A**) *S. aureus* growth under PG050 concentrations. (**B**) *S. aureus* growth under LE050 concentrations. (**C**) *E. coli* growth under PG050 concentrations. (**D**) *E. coli* growth under LE050 concentrations. (**E**) *P. aeruginosa* growth under PG050 concentrations. (**F**) *P. aeruginosa* growth under LE050 concentrations. (**G**) *B. subtilis* growth under PG050 concentrations. (**H**) *B. subtilis* growth under LE050 concentrations. (**I**) *S. enterica* growth under PG050 concentrations. (**J**) *S. enterica* growth under LE050 concentrations. (**K**) *Y. enterocolitica* growth under PG050 concentrations. (**L**) *Y. enterocolitica* growth under LE050 concentrations. (**M**) *L. monocytogenes* growth under PG050 concentrations. (**N**) *L. monocytogenes* growth under LE050 concentrations.

In order to assess the antibacterial effect of the polyphenolic extracts, half maximal inhibitory concentration (IC50), inhibitory concentration 90 (IC90), and minimum inhibitory concentration (MIC) were determined. Bacterial growth inhibition in the presence of extract concentrations is shown in Figure 3, whilst Table 1 displays IC50, IC90 and MIC values, expressed as a percentage of extract (*v*/*v*) in the incubation medium.

Significative antimicrobial differences were achieved (*p* < 0.05) between the tested extracts, being LE050 the one with the best inhibition values, especially related to the IC50 percentages. Nevertheless, both extracts showed antimicrobial activities, with remarkable effect against the most spread foodborne pathogens, *S. enterica*, *L. monocytogenes*, and *Y. enterocolitica*. A pattern of action was identified between Gram-positive and Gram-negative bacteria, being Gram-negative bacteria more sensitive to the extract, suggesting that the antimicrobial activity may be directly related to the bacterial wall composition. This result is supported by the nourishing effect observed for the PG050 extract on some Gram-positive bacteria, such as *B. subtitlis* (Figure 3G) and *L. monocytogenes* (Figure 3M), especially at high concentrations (5–10%). LE050 also shows *B. subtilis*’ growth enhancement at low concentrations prior to complete growth inhibition at 2.5% of the extract. However, different results were obtained among different bacteria and extracts suggesting that the extracts may have different antimicrobial strategies and targets.

**Figure 4:**
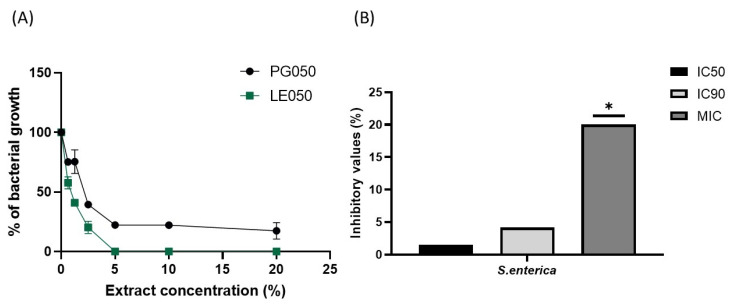
(**A**) Comparative growth of *S. enterica* growth in the presence of LE050 and PG050 extracts. A bactericidal effect was observed with LE050 extract, whereas a bacteriostatic activity was detected with PG050 extract at the tested concentrations. (**B**) Comparison of IC50, IC90, and MIC values obtained for *S. enterica* when using the PG050 extract. The closeness between IC50 and IC90 values is related to a bacteriostatic effect. * >20.

A bacteriostatic effect has been observed in PG050 *S. enterica* assay, where cell viability was compromised but a complete growth inhibition was not achieved. Bacterial growth tendency comparison between both extracts against the aforementioned pathogen is summarized in Figure 4A. The relation between IC50, IC90, and MIC values of *S. enterica* collected in Figure 4B where closeness indicates that this extract is bacteriostatic at the assessed concentrations.

In order to discard that the antimicrobial effect could be due to the solvent itself, controls using a hydro-organic mixture of both ethyl lactate and propylene glycol at the estimated concentrations present in the extracts (20:80 solvent: water) were performed. The results displayed on Table A1 (Appendix A) indicate that the solvents have a slight antimicrobial effect, not significative when compared with the extracts.

### 2.4. Inhibition of Biofilm Formation

The most common biofilm-formation bacteria were selected for the biofilm-inhibition assay. The capacity of the pathogenic strain to form biofilm was quantified by the crystal violet method. The results showed different effects of the extract on the growth of the biofilm. Results are presented in Table 2 as minimum biofilm inhibition concentration (MBIC) which was defined as the minimum concentration of extract that completely inhibits biofilm formation. Control assays for biofilm dispersion using hydro organic mixtures of the solvents (20:80 solvent: water) were performed, and no biofilm dispersion was achieved at the tested concentrations. Also, no biofilm eradication was observed with PG050 extract for *S. aureus* and *P. aeruginosa*, but 20% of the extract was enough for the complete dispersion of *L. monocytogenes*’ biofilm. On the contrary, quite effective biofilm inhibition was observed with concentrations of LE050 extract ranging from 0.625% to 2.5%.

**Figure 5:**
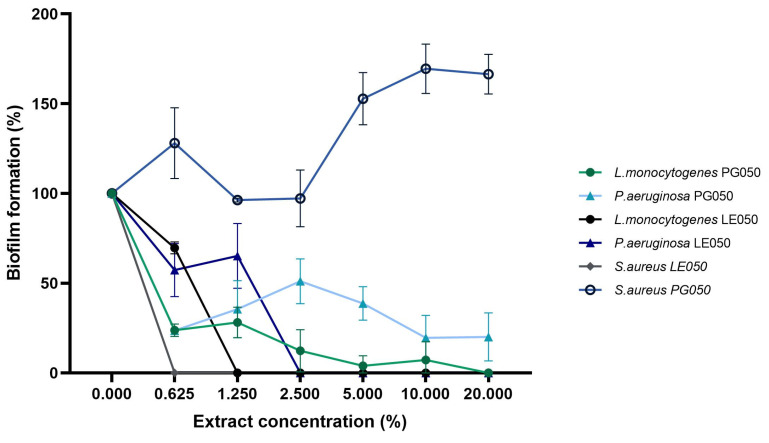
Biomass of biofilm produced by *L. monocytogenes*, *P. aeruginosa,* and *S. aureus* in the presence of different concentrations of the polyphenolic extracts. Bacterial biofilm formation assay was assessed for 72 h under biofilm desirable conditions and biomass measurement was performed by crystal violet biofilm interaction. Data are expressed as percentages of biofilm formation in the presence of different concentrations of the extract with respect with the biofilm formation in the absence of extract as means and standard deviation of the assays.

Biofilm formation of the tested bacteria was monitored under a wide range of extract concentration as shown in Figure 5. Under these growth conditions diverse effects on biofilm accumulation were observed. An increase in the biofilm quantification was seen on *S. aureus* under all the PG050 concentrations assayed. This result is consistent with the high IC50/MIC values observed for this combination, suggesting that the extract is not only having no antimicrobial effect on this pathogenic strain, (Figure 3A) but it also triggers its biofilm formation. *C. scoparius*’ PG050 extract seems to reduce biofilm accumulation of *P. aeruginosa,* although no complete elimination was observed at the concentrations tested. Nevertheless, 50% reduction was achieved at concentrations of the extract under 2.5%. In contrast to PG050, LE050 inhibitory effect was observed at the lowest concentration tested and the complete eradication was achieved at very low concentrations, suggesting that the distinct polyphenolic composition of both extracts is involved in bacterial biofilms inhibition.

## 3. Discussion

The study of natural products as antibiotic adjuvants and as antimicrobial alternatives has been increasing over the years in order to contribute to fight the worrying spread of superbugs and other emerging resistant strains. One of the areas where the transference of microorganisms commonly occurs is the food industry. Not only the manufacturing processes but also the livestock industry are affected by superbugs and foodborne pathogens [27].

An important element related to bacterial resistance, especially in food factories, is their capacity to form biofilms. These external structures that protect bacteria from external biocides are composed of planktonic and aggregated cells that act as a bacterial matrix endowing bacteria with adhesive and protective skills. These structures are quite difficult to disrupt and fast colonization, maturation, and spreading occur on equipment and facilities of the food manufacturing industry, which act as surface substrates [28].

The main objective of this research was to evaluate the potential bioactivity as antimicrobials of two rich polyphenolic extracts obtained from the shrub *C. scoparius*. In order to characterize their effectiveness against food pathogens and their suitability to be used in the food industry for surface decontamination, bacterial biofilm inhibition assays were performed.

The *C. scoparius*’ extracts used in this work present an elevated concentration of polyphenols, with TPC values much higher than the ones present in the extracts of other plants, such as *Capsicum lanceolatum* (250 mg GAE/L) or *Humbertia ambavilla* (100 mgGAE/L) [29]. The total polyphenolic content of LE050 extract is almost five times higher than that exhibited by PG050 extract, and there are also remarkable differences in their antioxidant activity, being the activity of EL050 extract twice higher than the one of PG050 extract. The extracts’ characterization by LC-MS/MS provided different polyphenolic profiles depending on the polarity of the solvent used, since the solvent allows the selective extraction of different molecules. LE050 extract contains twenty-three of the twenty-four polyphenols present in the scrub. These polyphenols are mostly classified as flavonoids and hydroxybenzaldehydes. On the other hand, PG050 extract presents lower total polyphenolic content, and during its individual polyphenolic characterization, a lack or a very low concentration of some of the polyphenols detected in LE050, such as 3-5 dymethoxybenzaldeyde, daidzein, 3-4-5 trimethoxycinnamic and apigenin, was observed.

However, PG050 extract contains a higher relative amount of phenolic acids, hydroxybenzoic acids being the most abundant, in comparison with LE050. Figure 2 summarizes *C. scoparius’* extracts relative composition. This polyphenolic characterization remarks the importance of a correct solvent selection in order to secure the best polyphenol bioaccessibility. Hence, polyphenols’ bioaccessibility is directly related to the extract’s bioactivity. LE050 extract, which possesses a higher total polyphenolic content and variety respect to PG050 extract, has also shown better efficiency as a pathogen controller.

Data shown in Figure 3 collect the growth behavior of the food pathogenic bacteria tested in the presence of different concentrations of the *C. scoparius’* extracts. Remarkable differences in the antimicrobial activity were assessed between both extracts. Nevertheless, a decrease in bacterial viability was observed on most of the assays performed, except for *Bacillus subtilis* assays, where an increase in the bacterial population was achieved at low-medium extracts’ concentrations. This nourishing behavior of the extracts was also observed in the *Staphylococcus aureus* PG050 assay and was especially reflected during its biofilm formation performance. Natural extracts are not only composed of polyphenols or antimicrobial substances; fatty acids, proteins, and sugars are also present [30,31,32]. These extra compounds can also play an important role in the bacterial metabolism, and at low concentrations, where polyphenol proportions are still low, they may even enhance their growth.

The different values of IC50 and MIC obtained for the antimicrobial assays also support the main role that solvents play in natural extract production. LE050 extract has IC50 values always below 3%, and it is quite effective against *Yersinia enterocolitica*, *Salmonella enterica* and *Listeria monocytogenes*, three of the most spread foodborne pathogens and those with the highest survival rates in different conditions [33]. Nevertheless, the values of IC50 obtained with PG050 extract are for most strains over 10%, and the MICs are also higher for this extract. IC90 values obtained for *S. enterica* (Figure 4A,B) reveal that this extract seems to have a very efficient bacteriostatic role against this pathogen, although it is inefficient for its complete elimination.

Previous studies performed by our group using natural products have shown the efficiency of polyphenols as antimicrobial, antiparasitic, and antifungal agents [34]. Recently, Guo et al. tested an olive oil polyphenolic extract against *L. monocytogenes* and suggested that polyphenols are involved in cell membrane depolarization and protein synthesis reduction [35]. With respect to the polyphenol composition-related efficiency, Zhao et al. proved the excellent antimicrobial activity of 3,4,5-trimethoxycinnamic and its derivates [36], these results indicating that this hydroxycinnamic acid may be responsible for the antimicrobial activity exhibited by our *C. scoparius* LE050 extract. Moreover, apigenin [37] and methoxybenzaldehydes [38], both polyphenols present in LE050 extract but absent in PG050 extract, have been reported to have activity against *S. aureus* and *E. coli*.

Both our extracts show better antimicrobial activity against Gram-negative bacteria, such as *E. coli*, *P. aeruginosa*, *S. enterica,* and *Y. enterocolitica*. Gram-negative bacteria superbugs are a major concern problem, because of their nosocomial fast spread and their resistance to a wide range of last-resort antibiotics. Different results were obtained by [26] Wassila Benabderrahmane et al., where a *Cytisus triflorus* ethyl acetate extract was assessed. These results indicate that both, the choice of solvent and the extraction method play a pivotal role in the antimicrobial activities, which proves that even very similar sources of polyphenols can yield marked different outcomes.

In order to explore the mechanism of action of the extract, the antibiofilm activity was evaluated. Figure 5 shows the end-point biofilm eradication results. *S. aureus*, *L. monocytogenes,* and *P. aeruginosa*’s biofilm formation were assessed over a wide range of extract concentrations independently of their IC50 and MIC antimicrobial values. Those results, especially the biofilm dispersion capacity of the LE050 extract, in combination with the growth tendency of the bacteria in the presence of the extract during the in vitro antimicrobial assays, suggest that biofilm inhibition occurs independently of planktonic cell viability. Similar results were obtained by [39] Audrey Charlebois et al. during their evaluation of *Clostridium perfringens* antibiofilm study, where they observed the independence of results for planktonic and biofilm cells during biofilm formation. Previous polyphenol antibiofilm assays against nosocomial pathogens detected biofilm inhibition related to a decrease in cell adhesion [40]. *S. aureus’* LE050 biofilm inhibition assay suggests that biofilm organization and synthesis stop at very low extract concentrations where bacterial viability was not affected, proposing that the extract may be involved in biofilm gene expression and quorum sensing [41]. PG050 extract shows lower efficiency on biofilm dispersion in comparison with LE050 extract, but the complete or almost complete dispersion was assessed at medium-high extract concentrations, around 10–20%. A totally opposite effect was observed on *S. aureus* PG050 biofilm evaluation, where an increase in the biofilm biomass was achieved. This is consistent with the results of the cell viability tests (Figure 3A) where apparently no antimicrobial effect of the extract was observed on these pathogenic bacteria. The fact that the oligosaccharides and other nutraceutical properties of the extract could nourish bacterial growth during the three days of biofilm formation could be related to this biofilm biomass increase in comparison with the rest of the bacteria and extracts. No growth effect was observed on *L. monocytogenes* and *P. aeruginosa*, suggesting that this biofilm enhancement is PG050 extract-*S. aureus* dependent. Similar nourishing results were obtained by Vladimir Plyua et al. during their evaluation of diverse phenolic acids against *P. aeruginosa* biofilm formation [42].

The present results obtained from the characterization of the *C. scoparius* extracts suggest that both extractants and bacterial strains play a main role in the antimicrobial effectivity. Antimicrobial and antibiofilm results obtained, especially for LE050 extract, suggest that natural extracts are valid agents to be incorporated into the food industry as foodborne controllers.

## 4. Materials and Methods

**Figure 6:**
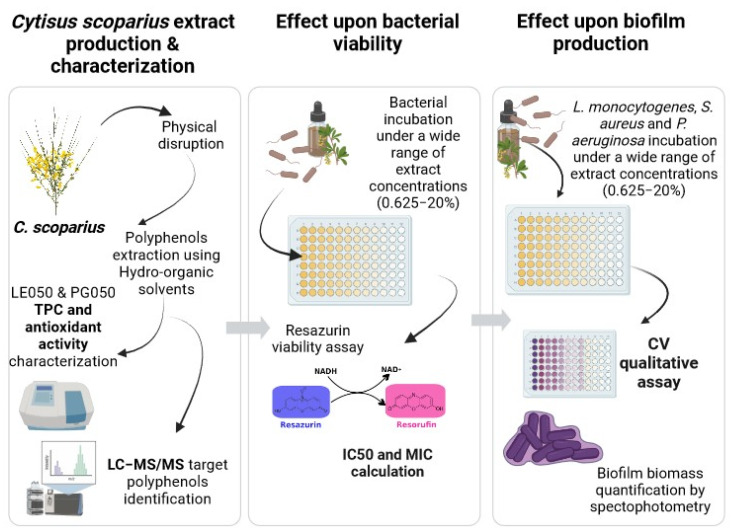
Diagram summarizing the methodology followed for the *C. scoparius’* extract production, analytical characterization, and bioactivity validation.

*C. scoparius’* extract production, analytical characterization, and bioactivity validation workflow is summarized in Figure 6.

### 4.1. Material

Propylene glycol and ethyl lactate (Scharlab, Barcelona, Spain) were used as extractive solvents. Polyphenol standards were purchased from Fluka Chemie GmbH (Steinheim, Germany) as previously described by Lores et al. [23]. The Folin–Ciocalteu phenol and Trolox reagents were obtained from Sigma-Aldrich (Steinheim, Germany). Sodium carbonate (Na_2_CO_3_, Panreac, Castellar del Vallès, Barcelona, Spain). Then, 2,2-diphenyl-1-picrylhydrazyl (DPPH, Tokyo Chemical Industry, Tokyo, Japan). Methanol and formic acid were supplied by Sigma-Aldrich and Merck (Darmstadt, Germany), respectively. Bacterial culture media, TSA, and BHI were purchased from (Condalab, Madrid, Spain). Cation Adjusted Müller Hinton II broth (CAMBH) from Becton-Dickinson (BBL, Sparks, NV, USA). Fetal bovine serum was supplied by Sigma-Aldrich, St. Louis, MO, USA. AlamarBlue from ThermoFisher Scientific in Waltham, MA, USA was employed as resazurin cell viability enzymatic substrate.

### 4.2. Medium-Scale Ambient Temperature Extract Production

The *C. scoparius* LE050 and PG050 extracts were obtained by MSAT system, under a patented procedure [43].

*C. scoparius* samples were collected from Santiago de Compostela, Spain, during spring. The samples were air-dried for several days in a cool, dry location at room temperature. For the MSAT procedure, 200 g of frozen scrub were crushed under mechanical grinding until a homogeneous particle diameter (about 5 mm) was obtained. The disruption was then dispersed with 250 g of sand using a mortar. Then, the mixture was packed on a glass column (23 cm × 50 mm Ø) with a 0-pore filter plate (160–250 mm) containing 1 g of sand layer at the bottom. Finally, the extracts were eluted with a hydro-organic mixture 1:1 of ethyl lactate—water for LE050 extract and propylene glycol—water for PG050 extract with a controlled extractive flow of 2 mL min^−1^.

### 4.3. Extract Characterization

To determine the solid content of the extracts, 2 g of each one was applied on metal assay plates in the moisture analyzer (Adam Equipment, Milton Keynes, UK, PMB 163). The analysis was carried out by gradually increasing the temperature to 110 °C and holding it stable for an average time of 20 min. The analysis was concluded when the change in the mass of the sample due to moisture, over a period of 1 min, was less than 0.001 g. The solid content is expressed as the percentage ratio m0 − mf/m0, where m0 represents the initial mass value and mf is the sample mass after moisture removal. All analyses were performed in triplicate.

Water content determination was carried out by Karl-Fischer HI933-02 analyzer (Hanna Instrument S.L., Gipuzkoa, Spain). Methanol extra dry was used as solvent and Karl Fischer’s reagent (Hydranal-composite-5) as titrant. Each liquid sample was accurately weighed in a syringe (50 mg) and introduced into the flask of the apparatus. The sample was shaken at 15,000 rpm for 1 min and titrated with the corresponding reagent. The endpoint criteria were set at drift stabilization (5 μg H_2_O min^−1^) or maximum titration time (10 min). Each sample was analyzed in triplicate.

### 4.4. Total Polyphenolic Index of the Extracts (TPC)

The *C. scoparius* extracts were assessed for their total polyphenolic content (TPC) using the Folin–Ciocalteu method, following the guidelines outlined by Rubio L. et al. [44]. Microtitration was conducted in 96-well plates with a microplate reader (BMG LAB-TECH, Ortenberg, Germany). In brief, 20 µL of the extracted substance was diluted and mixed with 100 µL of Folin–Ciocalteu reagent (1:10, *v*/*v*) and 80 µL of sodium carbonate solution (7.5 g L^−1^). The mixture was shaken and incubated in darkness for 30 min. Subsequently, the absorbance was measured at 760 nm. The TPC index was quantified using calibration curves of gallic acid across a concentration range of 20–160 mg L^−1^. TPC was expressed as milligrams of gallic acid equivalent per liter of extract (mg GAE L^−1^).

### 4.5. Antioxidant Activity of the Extracts

The antioxidant activity (AA) of the extracts was determined using the DPPH reagent following the method described by Castillo et al. [31]. Eight different concentrations of the extracts were mixed 1:1 *v*/*v* with 100 mL of DPPH reagent prepared in methanol. The mixtures were placed in 96-well plates and kept under dark conditions for 10 min. The absorbance was measured at 515 nm. The antioxidant activity (AA) was quantified using a calibration curve of Trolox spanning the range of 3–31 mg·L^−1^ (0.200–0.800 AU). The AA was expressed as millimoles of Trolox equivalent per liter of extract (mmolTE·L^−1^).

### 4.6. Characterization of Individual Polyphenols by Liquid Chromatography Coupled to a Tandem Mass Spectrometer (LC-MS/MS)

The quantification and characterization of individual polyphenols in the extracts were conducted using LC-MS/MS with a Thermo Scientific instrument based on a TSQ Quantum UltraTM triple quadrupole mass spectrometer. Optimal instrumental conditions were previously optimized by Celeiro et al. to obtain the best chromatographic separation of the target polyphenols [45]. The chromatographic separation utilized a Kinetex C18 column (100 mm × 2.1 mm, 100 Å) from Phenomenex (Torrance, CA, USA). The mobile phase consisted of water (A) and methanol (B), both with 0.1% formic acid. The chromatographic gradient was set at 5% B, reaching 90% B in 11 min and maintained for 3 min, with initial conditions achieved in 6 min. The injection volume was 10 µL, and the flow rate was 0.2 mL min^−1^, with a column temperature of 50 °C. Compound identification and detection were performed by selected reaction monitoring (SRM) working simultaneously in both negative and positive modes, monitoring two or three MS/MS transitions for each compound. The system was operated using Xcalibur 2.2 and Trace Finder 3.1 software. External calibration was used for the quantification of polyphenols. Linearity was evaluated in a wide range of concentrations from 0.01–10 μg mL^−1^, employing standard solutions prepared in water/methanol (50:50 *v*/*v*). The obtained coefficients of determination (R^2^) were, in all the study cases, higher than 0.9900.

### 4.7. Bacterial Strains and Culture

Antimicrobial activity of the extracts was assessed against some general pathogenic bacteria. *Escherichia coli* ATCC 25922CECT and *Pseudomonas aeruginosa* ATCC 27853 as representative Gram-negative strains and, *Staphylococcus aureus* ATCC 25923 and *Bacillus subtilis* ATCC 6633 as Gram-positive ones. In order to prove their potential application as foodborne controllers, the extracts were also assayed against the three most world-spread alimentary pathogens; *Listeria monocytogenes* CECT 4032, *Yersinia enterocolitica* and *Salmonella enterica subsp. Enterica* CECT 554.

The *Y. enterocolitica* strain used in this study is a clinical fecal isolate from the Microbiological laboratory of Complejo Hospitalario Universitario de Ferrol (Ferrol, A Coruña, Spain).

Strains of *E. coli, P. aeruginosa, S. aureus, L. monocytogenes, Y. enterocolitica* and *S. enterica* from the frozen stock (−80 °C) were seeded on TSA (Triptone soja agar) medium plates and incubated for 24 h at 37 °C. BHI-agar medium (Brain heart infusion agar) was used to grow *B. subtilis* at 37 °C for 24 h.

### 4.8. Determination of the Antibacterial Activity by the AlamarBlue Viable Cell Count Method with Fluorometric Reading

In order to determine the antibacterial activity of the extracts viable, cell account by fluorometric reading was used. EUCAST inhibitory assay considerations were followed with slight modifications that allow to explore the extracts’ effect either as bactericidal or bacteriostatic compounds. Briefly, 100 μL of a bacterial concentration of 10^6^ colony forming units (CFU)/mL in Cation Adjusted Müller Hinton II broth (CAMBH, Camden, NJ, USA) was mixed with 40 μL of each extract concentration assayed (0%, 0.625%, 1.25%, 2.5%, 5%, 10%, and 20%) on a 96-well microplate. Saline Phosphate buffer (PBS 1M) was added to the mixture to control pH variation to a final volume of 200 μL. The microplate was incubated for 21 h at 37 °C. In order to assay the bacteria at the optimum growth conditions, *P. aeruginosa* CAMBH was supplemented with 2% fetal bovine serum (FBS). A blank of the extract was employed by incubating 100 μL of CAMH broth instead of the bacterial inoculum. After the incubation, 100 μL of fresh culture broth, 60 μL of phosphate-buffered saline (PBS, 1M), 20 μL of alamarBlue (from ThermoFisher Scientific in Waltham, MA, USA), and 20 μL of each well from the overnight incubated plate were mixed in a new 96-well microplate in order to assess at which concentrations the activity is bactericidal or bacteriostatic. The absence of growth in the second plate indicates that no cells remain viable after the incubation and hence the extract at that concentration is bactericidal, whereas growth in the second plate with a clear depletion in the number of cells indicates that the extract acts as a bacteriostatic at that concentration. Fluorometric reading was performed to determine the number of viable cells. AlamarBlue resazurin was used as an enzymatic substrate. Fluorescence assays were conducted with measurements taken at an excitation wavelength of 544 nm and emission wavelength of 590 nm, utilizing the FLUOstar microplate reader (BMG Labtech, Ortenberg, Germany). The experiments were performed in triplicate and repeated three times.

### 4.9. Inhibition of Biofilm Formation

Biofilm inhibition assay was performed following the protocol of Wilson et al. for qualitative biofilm measure by crystal violet [46]. *L. monocytogenes*, *P. aeruginosa*, and *S. aureus* were chosen as foodborne potential biofilm producers. Briefly, bacterial cultures were exposed to different extract concentrations as described above for the viability cells assay. Cultures were kept for 72 h at 37 °C in order to allow bacterial biofilm formation on the 96-microwell plates. After biofilm formation plates were washed with distilled water twice in order to eliminate planktonic cells. Then, 100 µL of a 1% crystal violet solution was added to the plates and room temperature incubation was performed for 20 min. After incubation, the dying solution is removed, and the biofilm is thoroughly washed multiple times with distilled water to eliminate any residual free dye. After that, 90% ethanol was used as decoloring solution. 200 µL of decoloring solution was added to the biofilm to solubilize it and 10 min incubation occurs. Finally, absorbance was measured at 570 nm with a multi-well plate UV-Vis spectrometer.

MBIC extract concentrations were calculated as follows:

$$MBIC=\frac{Concentration\;of\;biofilm\;measured\;at\;X\;extract\;concentration}{Concentration\;of\;biofilm\;measured\;at\;control}\times 100$$

### 4.10. Statistical Analysis

Individual polyphenols characterization was expressed in a relative concentration of normalized values. Data were expressed as means ± SD. Statistical differences were analyzed using the Two-ways ANOVA with Graphpad Prism 9.0. The differences were considered statistically significant at *p* < 0.05. Extract inhibitory values, IC50 and IC90 were calculated by Graphpad Prism 9.0. IC50, IC90, and MIC values are expressed as means ± SD of the quantified data. Statistical differences were calculated by one-way analysis of variance (ANOVA).

## 5. Conclusions

*C. scoparius* polyphenolic extracts show a diverse and promising antimicrobial and antibiofilm activity. The selected solvents—propylene glycol and ethyl lactate—both classified as GRAS, performed an efficient extraction of polyphenols with diverse bioaccessibility. Different bioactivity efficiencies were recovered for each combination extract- pathogen, suggesting that the synergistic effect of the extract’s polyphenols interact in different manners with different bacterial strains. The different pattern of action of the antimicrobials was possibly due to the variety of mechanisms of action of polyphenols, their synergy, and the complexity of the extracts. Despite this, the most effective antimicrobial activity was obtained for LE050 extract against gram negative foodborne pathogens. 

Biofilm formation and dispersion assays suggest that *C. scoparius* polyphenolic extracts may be involved in Quorum sensing and biofilm inhibition pathways. Both extracts have shown a strong reduction in biofilm accumulation for the main foodborne pathogens, especially *L. monocytogenes*. PG050 extracts seem to act as bacteriostatic, rather than bactericidal at the studied concentrations and even its composition seems to enhance bacterial growth at low concentrations.

Future studies should be performed in order to elucidate the mechanisms of action of *C. scoparius* polyphenolic extracts on cell viability, as well as their role in biofilm inhibition and Quorum sensing signaling.

## Figures and Tables

**Table 1:** Antimicrobial activity of the extracts expressed as IC50 (extract’s concentration needed for 50% inhibition of bacterial growth), IC90 (extract’s concentration needed for 90% inhibition of bacterial growth), and MIC (minimum extract concentration needed for inhibiting completely bacterial growth). All experiments were performed in triplicate.

Strain	LE050			PG050		
	IC50	IC90	MIC	IC50	IC90	MIC
*Escherichia coli*	2.09	2.79	10	18.12	20	≥20
*Staphylococcus aureus*	3.27	5.19	10	19.5	20	≥20
*Pseudomonas aeruginosa*	1.41	2.43	5	12.03	20	≥20
*Bacillus subtilis*	1.93	2.53	2.5	19.44	20	≥20
*Salmonella enterica*	1.2	3.55	5	1.48	4.15	≥20
*Listeria monocytogenes*	3	4.37	5	20	20	≥20
*Yersinia enterocolitica*	0.63	1.21	2.5	4.87	5.57	10

**Table 2:** MBIC values. Percentage of the extract that completely disperses the pathogens’ biofilms. nd refers to a non-complete dispersion. Tests were performed in triplicate and three times.

Strain	LE050 (%*v*/*v*)	PG050 (%*v*/*v*)
*Staphylococcus aureus*	0.625	nd
*Pseudomonas aeruginosa*	2.5	nd
*Listeria monocytogenes*	1.25	20

## Data Availability

Data will be available on request to corresponding authors.

## Appendix A

**Table A1:** Antimicrobial activity of the solvents used for the extraction process at the estimated concentrations present in the final extracts. Data are expressed as IC50 (extract’s concentration needed for 50% inhibition of bacterial growth), IC90 (extract’s concentration needed for 90% inhibition of bacterial growth) and MIC (minimum extract concentration needed for completely inhibiting bacterial growth). All experiments were performed in triplicate.

Strain	LE050			PG050		
	IC50	IC90	MIC	IC50	IC90	MIC
*Escherichia coli*	12.63	20	>20	20	>20	>20
*Staphylococcus aureus*	6.89	20	>20	9.37	>20	>20
*Pseudomonas aeruginosa*	5.36	13.13	20	>20	>20	>20
*Bacillus subtilis*	4.77	5.86	10	>20	>20	>20
*Salmonella enterica*	1.58	2.75	10	1.65	20	>20
*Listeria monocytogenes*	3.05	5.12	10	>20	>20	>20
*Yersinia enterocolitica*	1.37	1.84	5	4.38	20	>20
